# Cognitive Control of Eating: the Role of Memory in Appetite and Weight Gain

**DOI:** 10.1007/s13679-018-0296-9

**Published:** 2018-02-12

**Authors:** Suzanne Higgs, Maartje S. Spetter

**Affiliations:** School of Psychology, University of Birmingham, Edgbaston, Birmingham, B15 2TT UK

**Keywords:** Episodic memory, Working memory, Eating behaviour, Obesity, Cognition, Appetite

## Abstract

**Purpose of review:**

The present review organises the recent literature on the role of memory in eating behaviours and provides an overview of the current evidence relating to the associations between memory and weight gain.

**Recent findings:**

Research over the last few years has highlighted working memory as an important cognitive process that underpins many aspects of appetite control. Recent work on episodic memory and appetite has replicated work showing that manipulating memory for recent eating affects later consumption and extended this work to examine associations between individual differences in memory and eating behaviours. Poorer episodic memory ability is related to a reduced sensitivity to internal states of hunger and satiety and a tendency towards uncontrolled eating. There is also recent evidence to suggest that working memory and episodic memory impairments are related to weight gain and high BMI.

**Summary:**

Working memory and episodic memory are core cognitive processes that are critical for food-related decision-making, and disruption to these processes contributes to problems with appetite control and weight gain, which suggests that weight loss programmes might be improved by the addition of cognitive training.

## Introduction

The importance of cognition in the control of eating has been recognised for some time, notably by Stanley Schachter who found that manipulating cognitions present at the time of an eating episode affected the food intake of both lean and higher weight participants [1]. More recently, there has been interest in identifying the specific cognitive processes that underpin food-related decision-making [2]. Higgs and colleagues reviewed the literature on the role of learning and memory in the control of eating and highlighted working memory and episodic memory as key processes [3]. The aim of the present review is to provide an update on recent research into cognitive controls of eating, with a specific focus on memory processes in humans. We will discuss food choices in the context of current neuro-cognitive models of decision-making and highlight how metabolic factors interact with these decision-making processes. We then focus on reviewing the evidence that working memory and episodic memory processes are associated with eating behaviours. We will also examine recent research that has suggested that disruption to these memory processes is associated with overeating and weight gain. We will conclude by briefly discussing the implications of research in this area.

## Cognitive Processes Underpinning Eating Behaviours

To make a food choice, we must first recognise an object as food [4]. Hence, the first cognitive process that underpins the control of eating behaviour is the detection and processing of a relevant stimulus (in this case food). Imagine walking into your kitchen and seeing a packet of cookies next to the fruit bowl on the counter, whether you decide to open the cookie packet or take an apple from bowl will first depend on whether you notice there is food there at all. Once a food has been attended to and recognised as a potential object of desire, in that moment of decision-making, the brain will be performing a range of computations that may result in an action being taken (e.g. picking the apple) or an action being withheld (e.g. resisting taking the cookie). Let us say your attention is drawn (more) to the cookies, you might favour opening the packet over reaching for the apple, but whether you do so will depend on a number of factors: whether you have eaten recently, if you enjoyed eating those particular cookies before, whether taking the cookie is consistent with your current eating and health goals, if there is someone else with you at the time and whether it is usual that you take a cookie when you enter the kitchen. Therefore, eating decisions are the outcome of a cognitive process that involves integrating a range of inputs in memory, including sensory, somatic, affective, socio-cultural and contextual information [5]. Once the decision is made to eat (or not), then the outcome of that decision will be updated in memory; did the cookie taste as expected? Did it deliver energy? This information will be used to inform future decision-making, for example by updating information stored in memory.

## Specific Cognitive Control Systems

Research into the neuro-computational basis of decision-making has identified specific types of computations that take place before, during and after a choice that can be applied to cognitive control of eating (for a review see [6]). The sight of a packet of cookies may evoke a desire to grab a cookie because we have previously learned that the taste of cookies is associated with a positive hedonic response (this is known as Pavlovian learning) (for a review see [7]). In addition to learning about associations between a stimulus (cookie) and a response (pleasure of eating), we also learn associations between actions and the consequences of actions: the act of eating the cookie becomes associated with obtaining a pleasurable reward (the cookie) (this is known as instrumental or goal-directed learning) (for a review see [8]). A consequence of this type of learning can be that with repeated experience, the action may be elicited in the presence of associated contextual cues regardless of the outcome: in other words, the behaviour becomes habitual. An example would be reaching for the cookies even though we are trying to avoid eating high-calorie snacks. This is because habitual behaviour is guided by stored information about the average immediate consequences of performing the action and so is not sensitive to representations of delayed outcomes, such as the knowledge that eating the cookie would not be consistent with our current long-term health goals. Of course, we are capable of responding to the sight of the cookies based on abstract representations of outcomes, and this is what is known as goal-directed behaviour. Goal-directed behaviour is flexible because the action selected at any decision point is based on a mapping of many possible outcomes (even outcomes that have yet to be experienced) and their current expected value. In this case, the decision about whether to take a cookie, or an apple, or no food at all, is based on computing the overall value of that action, taking into account a range of potential outcomes that could include the expected taste of the food, the delayed impact of eating the food on health and/or the social consequences of eating at that moment.

At a particular moment, behaviour may be guided primarily by Pavlovian, habitual or goal-directed control processes and which type of control predominates will depend upon the specific context in which the choice is being made and the perceived potential benefits of adopting one type of strategy over another. Habitual control may be favoured as a computationally efficient strategy, but in unfamiliar situations, goal-directed processes are likely to predominate over habitual processes [8]. In some situations, there may be conflict between the actions favoured by different control systems. For example, habitual control might promote taking the cookie but the goal-directed system might favour taking the apple instead; in addition, both processes have been associated with different reward circuits in the brain [9]. The extent to which behaviour is guided by the goal-directed, habitual or Pavlovian system could explain why some people find it easier to stick to diet goals than do others.

## Interactions with Metabolic Signals

Neuro-cognitive decision-making processes that underpin eating behaviour interface with the physiological systems that provide information about metabolic state and their processing in the brain (homeostatic systems): changes in metabolic state, such an energy deficit, motivate eating behaviour via modulation of cognitive processes (for a review see [10]). Current metabolic state biases responses to conditioned food cues: food cues are more salient and hedonic responses to food are enhanced when we are hungry [11, 12]. In addition, a state of nutritional repletion reduces the ability of food cues to excite the memory of the rewarding postingestive consequences of eating and so inhibits eating [13•]. It is also likely that metabolic signals act in the brain to alter goal-directed learning processes, for example by altering goal-directed values assigned to food or by affecting the learning of act-outcome relationships. Research into the precise ways in which metabolic signals may affect food-related decision-making in humans is growing and will yield important insights into how the ingestion of food and the associated satiety signals inhibit eating (for a review see [10].

## The Role of Memory

Memory processes are fundamental to food-related decision-making. For example, the associations between foods and representations of the outcomes of eating those foods that underpin conditioning are stored in memory. Working memory, e.g. holding information in mind and processing this knowledge is required for the integration of goal values and a number of recent studies have investigated the role of working memory processes in eating behaviours (for a review see [14]). The importance of episodic memory, which is memory for a specific event or episode, in relation to appetite has also been investigated (e.g. [15•]). Memory for recent eating (a type of episodic memory) has been highlighted as playing a role in satiety [16, 17]. Episodic memory may serve an important function in allowing us to use information about recent eating to predict future food needs and integrate this information with knowledge about current food availability to adapt motivation accordingly [18]. It has been suggested that impaired episodic memory ability may be related to feelings of uncertainty about the availability of food due to a reduced ability to envision and plan future eating episodes and food availability [19]; this insecurity can ultimately lead to an increase in food intake and therefore fat storage, which could increase the likelihood of obesity [20•]. It has been also argued that memory for individual eating events may be used in goal-directed computations to predict the outcome of a decision [2]. For example, in deciding whether or not to eat a cookie, we may factor into that decision the memory of the last time we ate that type of cookie and how much we enjoyed that experience.

## Working Memory and Eating Behaviour

Holding food-related information in working memory increases the attention paid to food cues: we pay more attention to food in our environment if we are already thinking about food, because attention is drawn particularly efficiently to an item that matches the representation in working memory [3, 21–23]. It has also been reported that maintenance of attention to food cues is linked to greater reinforcing efficacy of food and that this is enhanced in participants with obesity who have a high working memory capacity [24]. Attentional biases towards food have been linked to increased food intake and hunger [25], possibly because paying attention to a stimulus also increases the readiness to execute actions associated with that stimulus e.g. reaching for a tempting food [26]. In addition, paying attention to specific attributes of food such as their hedonic attributes may bias consumption choices because these attributes of food are given greater weight in goal-directed valuation processes [27].

Once attention has been directed towards food and the decision to eat has been made, then attention to food determines the subsequent rate of satiation and intake. Intake is increased if attention is drawn away from food as it is eaten by engagement in a secondary activity, such as watching TV [28]. These data suggest that the maintenance of food consumption information in working memory is a mechanism underpinning satiation. In support of this idea, it has been reported that participants with larger working memory capacity satiate faster to a variety of stimuli than do those with a lower working memory capacity [29••]. We have also reported that natural satiation increases activity in the dorsolateral prefrontal cortex (dlPFC) [30••] in response to food stimuli, an area of the brain that is associated with attention, memory and cognitive control [31]. Investigation of the specific role of working memory processes in satiation is an area for ripe for research.

Working memory also underpins the ability to maintain attention on current long-term goals [32]. A high working memory capacity enables individuals to maintain a focus on goals and facilitates actions that are consistent with these goals [33]. There has been some investigation of the relationship between working memory and food intake including four reports of a positive correlation between working memory and fruit/vegetable intake [34–37]. One study reported no relationship between working memory and fruit/vegetable intake, but here, a questionnaire-based measure of working memory was used rather than a behavioural measure [38]. There have been two reports of a negative correlation between working memory ability and snack intake [35, 39] and two reports of no relationship between WM and fat intake [34, 38]. These data suggest that working memory ability may be more important when making decisions about fruit and vegetable intake than when making decisions about snack foods/high energy dense food intake, which would be consistent with the idea that working memory may be critically deployed in decisions that involve taking the health consequences of eating into account. Indeed, working memory capacity may be a critical factor in helping people stick to their diet-related goals [32, 37, 40]. However, working memory has also been implicated more generally in behavioural control: high and low working memory function have been linked to excessive appetite control in anorexia on the one hand and lack of control over eating in binge eating disorder on the other hand [41, 42•, 43, 44]. Working memory likely underpins many of the processes that influence food decision-making and future work could usefully investigate when and how working memory processes are involved in food-related decision-making and how such processes may be altered in disordered eating.

## Episodic Memory and Eating Behaviour

The possibility that memory for the most recent eating episode plays a role in everyday decisions about eating was first investigated experimentally using a recall paradigm in which participants were provided with a fixed lunch in the laboratory, and then 2–3 h later, were asked to recall what they remembered about eating this meal [16, 45]. Recalling the lunch eaten earlier that day, but not a lunch eaten the previous day, reduced afternoon snack intake. The explanation offered was that memory of recent eating normally inhibits consumption and recalling the most recent episode boosted this effect. Recent findings have confirmed that recall of a recent eating episode inhibits later food intake, whereas recall of neutral information, an exercising episode, or lunch eaten the previous day, has no effect on intake [46–48]. It has also been reported that the effect of memory for recent eating is larger when recall takes place a few hours after lunch, than when it occurs only an hour later [45]. These data suggest that at least some forgetting of the meal is required before an effect of recall is evident, which is consistent with the idea that memory is an underpinning process.

Another way to test the idea that memory for recent eating inhibits later intake is to disrupt the encoding of the meal memory, for example, by distracting people from eating by asking them to engage with secondary activity such as watching TV or playing a computer game. In this case, one would predict a poorer memory of the meal because encoding would be disrupted by dividing attention between the meal and the secondary activity. Consequently, the inhibitory effect of memory for recent eating should be undermined and so participants should eat more when given the opportunity to snack later. Following the initial demonstration that people do indeed consume more afternoon snacks after a TV lunch than after eating the same amount of food when there was no distraction [49], the effect of distraction during eating on later consumption has been replicated many times [49–52]. Moreover, in all these studies, there was poorer recall of the amounts eaten in the distraction condition, suggesting that impaired memory of the recent eating episode was responsible for the enhanced intake. Stevenson and colleagues also recently observed that intake of a meal was higher after earlier snacking while watching TV than after snacking with no TV, but this was only the case for men [53]. These authors found no relationship between meal memory and later snacking, but the design of their study differed from previous studies in that the number of snacks eaten was not fixed (and so was not the same across the experimental conditions), which complicates interpretation of the findings.

If disrupting meal memory encoding impairs memory for recent eating, then asking people to pay more attention to food as it is eaten should facilitate meal memory encoding and reduce later food intake [54]. This possibility has been investigated in four studies [22, 55–57]. All studies found that focusing on food while eating was associated with reduced snacking later (the next eating episode), but no clear evidence has emerged that the effect is related to enhanced memory of the previous eating episode: only one of the studies [55] found evidence that focusing on food was associated with improved meal memory. It may be that ceiling effects prevented any effects of memory enhancement being detected, but it is also possible that the effect of “attentive eating” on later intake is not underpinned by changes in meal memory and this possibility should be explored. Overall, there is robust evidence that manipulating memory for recent eating by meal recall or distraction affects later intake, but the effect of eating more “mindfully” on memory is not clear at present.

A striking demonstration of the association between episodic memory and eating behaviours comes from studies of patients with damage to the hippocampus who have a deficit in encoding new information in memory and who also have disturbed appetite [58]. For example, amnesic patients will eat one meal after another meal without showing a decline in hunger [58, 59]. Three recent studies have investigated the links between episodic memory ability and eating behaviours in non-clinical samples. Stevenson and colleagues have reported that episodic memory is related to sensitivity to hunger/satiety cues [60]. Participants, who reported consuming a diet rich in fat and sugar had reduced performance on an episodic memory task, were less accurate in recalling what they had previously eaten and showed reduced sensitivity to internal signals of hunger and satiety, relative to a group consuming a low-sugar/fat diet [61••]. In another study by the same group, hippocampal-dependent memory performance was related to the reduction in desire to consume palatable food when sated [62]. Martin and colleagues investigated the relationship between episodic recall performance and self-reported eating traits [63••]. Participants who reported being more susceptible to uncontrolled eating exhibited poorer episodic recall, whereas participants who reported more successful control over their intake exhibited better recall [63••]. Taken together, these data provide further support for the suggestion that episodic memory processes are involved in the control of eating.

## Memory and Weight Gain

The involvement of working memory and episodic memory in the control of eating behaviours suggests that if these processes are disrupted, then problems with appetite control and weight gain should result. This proposition can be tested by examining whether memory problems are associated with weight, or more precisely, whether memory problems predict weight gain. There has been a large increase in the number of studies examining the relationship between BMI and cognitive function in the last few years. Here, the focus is on summarising the key advances in the area.

## Is There a Relationship Between Memory and BMI?

A systematic review of the relationship between cognitive function and obesity in adults conducted by Prickett and colleagues [64•] did not find consistent evidence that high BMI was associated with impairments in memory, although as the authors noted, various limitations to the studies reviewed mean that some caution is required in interpreting the findings. Most notably, the assessments of memory function were limited both in terms of the number of studies assessing memory and the specific types of memory that were assessed. Similarly, a systematic review of the relationship between cognitive function and obesity in children and adolescents revealed few studies that included specific assessments of memory [65]. The inconsistency of results across studies in this area may be explained by the differing assessments of memory used and the lack of systematic accounting for potential confounders of any relationship between obesity and memory function, such as comorbidities, socio-economic status, education and depression and the effects of stigma directed towards people with a high BMI on cognitive function.

The results of recent studies have provided more consistent evidence that high BMI is associated with memory impairments in adults. Cheke and colleagues reported that higher BMI was associated with lower performance on the what–where–when (WWW) task that assesses episodic memory, even after controlling for the effects of age, sex and years in education on performance [15•]. A further study by the same group examined neural activity in low versus high BMI participants while performing the WWW-task. High BMI participants had reduced activity in key memory structures, relative to low BMI participants, although this reduced neural activity was not reflected in a reduction in performance on the WWW-task [66]. Prickett and colleagues reported that obesity was predictive of poorer performance on both a verbal memory and a working memory task and this association remained after controlling for obesity-related comorbidities [67]. In addition, memory recognition (immediate and delayed) and digit span (forward and reverse) performance was reported to be lower in overweight and obese young women after controlling for confounders [68]. Overall, recent data indicates that there is an association between levels of body fat and memory performance. Future research may be directed at identifying moderating factors such as education level and/or cognitive reserve [69, 70].

The data from behavioural studies are consistent with the results of structural imaging studies, which show that a high BMI is associated with a reduction in grey matter volume (GMV) in brain in areas associated with memory, including the hippocampus, prefrontal cortex [71, 72, 73•, 74–76]. Reductions in white matter integrity and volume have also been reported [77, 78], especially in connections within the limbic system and those connecting the temporal and frontal lobes (for a review see [79]). In addition, there is evidence that BMI-associated structural alterations are related to memory problems; Masouleh and colleagues reported that reductions in GMV in frontal and thalamic brain areas mediated the relationship between BMI and poorer memory [80]. Two recent studies have assessed the relationship between resting state activity and BMI. Both studies reported reduced activity in the default mode network in participants with high BMI [81, 82], although the links to cognition were unclear and this could be a topic for further study.

Several recent studies have also provided evidence for reduced memory ability at higher BMI in children. Level of abdominal adipose tissue was negatively associated with hippocampal-dependent memory ability in one study of overweight and obese children aged 7–9 [83], which may relate to reduced hippocampal volume as has been found in adolescents with obesity [84]. Goldschmidt and colleagues reported an association between overweight and poorer working memory performance but only for those children who also reported loss of control eating [85].

An important issue to bear in mind when considering the results of studies on the relationship between BMI and cognition is that majority of studies have been cross sectional and, therefore, while it may be the case that memory problems contributed to weight gain, a causal mechanism cannot be inferred from such studies. The results of longitudinal studies of adults suggest that cognitive impairments may predate weight gain [35]. A 12-month longitudinal study of children aged 6–11 years revealed that working memory deficits are already present in young children. The ability to shift attention predicted BMI at time 2 whereas BMI at time 1 was not predictive of a change in cognitive performance at time 2, which is suggestive that impairments put children at risk of becoming overweight [86•].

Evidence from rodent models is consistent with the suggestion that deficits in memory leads to problem with appetite dysregulation, which leads to subsequent weight gain [87]. Nevertheless, other evidence indicates that it is just as likely that obesity results in cognitive problems (for a review see [88]), with a likely mechanism being the effects of obesity-related inflammation on the brain [89, 90]. In fact, it has been proposed that memory impairments might contribute to overeating and weight gain, and in turn, weight gain might lead to memory problems to perpetuate a vicious cycle [91].

There is evidence to suggest that memory impairments are associated with problems with appetite control, which are likely to contribute to weight gain, but the extent to which they associated with wider functional impacts is unclear. Indeed, many of the effects are subtle and it has yet to be established how much variance in eating behaviours and weight gain are accounted for by individual difference in memory. Studies assessing memory as a predictor of eating behaviours and body weight in a large population, while accounting for other established predictor variables, are needed to answer these questions.

## Implications for Weight Management

An important implication of the research outlined here is that improving memory function may be a useful adjunct to interventions aimed at improving appetite control. Indeed, it is well known that tracking food consumption (e.g. using food diaries) is associated with better weight outcomes and one reason why this might be the case is that tracking improves memory for recent eating [54]. There is promising evidence that the cognitive problems associated with obesity are be reversible; weight loss in people with obesity is associated with improvements in cognitive performance [92] and a change in diet from high-fat to a low-fat diet can reverse the neurological changes associated with the consumption of such diets in rats (see [93, 94], for a review of the effects of diet on cognition; [95]). Interest in developing cognitive training programmes to improve eating behaviours has increased recently [96–98]. Some programmes are aimed specifically at altering eating behaviour by training working memory [99] and meal memory encoding [100]. Many of these programmes are in the early stages of development and it remains to be seen whether they prove successful in large-scale trials. Key challenges are improving training adherence and transfer of the effects to behaviours outside of the laboratory, which may be addressed by technological innovations such as gamification, virtual reality and personalisation [101]. A potentially effective approach that deserves investigation is to combine cognitive interventions with existing dietary, pharmacological and surgical interventions for obesity.

## Conclusion

Eating behaviours are underpinned by neuro-cognitive decision-making processes that rely on working memory and episodic memory. These systems are influenced by metabolic signals that provide information about short- and long-term energy storage, thus providing a mechanism by which nutritional needs are linked to motivation and behavioural control. Disruption of memory systems is linked to appetite dysregulation and weight gain, which suggests that targeting memory processes may be beneficial in interventions aimed at promoting healthier consumption.
